# Piquin chili, a wild spice: natural variation in nutraceutical contents

**DOI:** 10.3389/fnut.2024.1360299

**Published:** 2024-04-15

**Authors:** Rogelio Pérez-Ramírez, Yolanda del Rocio Moreno-Ramírez, Gilberto Ruiz-De-La-Cruz, María Cruz Juárez-Aragón, César Leobardo Aguirre-Mancilla, Nohemí Niño-García, Jorge Ariel Torres-Castillo

**Affiliations:** ^1^Instituto de Ecología Aplicada, Universidad Autónoma de Tamaulipas, Ciudad Victoria, Tamaulipas, Mexico; ^2^Facultad de Ingeniería y Ciencias, Universidad Autónoma de Tamaulipas, Ciudad Victoria, Tamaulipas, Mexico; ^3^Laboratorio de Biotecnología Animal, Centro de Biotecnología Genómica, Instituto Politécnico Nacional, Reynosa, Tamaulipas, Mexico; ^4^Departamento de Posgrado, Instituto Tecnológico Roque, Tecnológico Nacional de México, Celaya, Guanajuato, Mexico; ^5^Unidad Académica Multidisciplinaria Mante Centro, Universidad Autónoma de Tamaulipas, Ciudad Mante, Tamaulipas, Mexico

**Keywords:** nutraceuticals, bioactive compounds, chili, capsicum, wild plants

## Abstract

The piquin chili is a wild spice widely consumed from the South United States to Central America and stands out as a source of flavonoids, essential metabolites with antioxidant properties. The concentrations of flavonoids, carotenoids, and capsaicinoids vary according to regions, maturity stages, and ripening processes. These compounds, which are known for their health benefits and industrial applications, highlight the importance of identifying ideal environmental conditions for collecting fruits with the highest contents. Comprehensive studies of the piquin chili are essential for understanding its properties for the benefit of consumers. This approach fortifies trade, contributes to resource conservation, and advances cultivated chili production.

## 1 Introduction

The plants synthesize phytochemical compounds as part of biosynthetic processes to perform ecological and physiological functions. The phytochemical compounds are perceived through aromas, flavors, and colors, which humans could exploit (1). The wide phytochemical diversity with nutraceutical potential from wild plant sources includes phenolics, alkaloids, terpenoids, polysaccharides, glycosides, and compounds of a peptide nature (2–4).

The consumption of nutraceutical sources from vegetal ecosystems is widely practiced across world cultures, showing the importance of conservation and proper management of natural resources (5–7). Nevertheless, consuming products with nutraceutical potential from wild sources must consider the differences in contents and composition compared with cultivated forms, which consequently affect the product quality, organoleptic, and functional characteristics, that are considered as influencing elements of consumer decision (8–11). It is imperative to perform comprehensive studies to maintain optimal production conditions for nutraceuticals, recreating natural processes supported by genetic improvement, analytical techniques, traditional practices, natural resource management, industrial process innovations, specific sustainable forestry production principles, and public policies for their exploitation (12).

The piquin chili, *Capsicum annuum* var. *glabriusculum* (Dunal) Heiser and Pickersgill, is a wild form of *Capsicum*, considered the ancestor of *anunum* species, widely distributed from the southeast of the United States of America to northern South America, with names such as piquin, chile de monte, chiltepin, chiltepe, amashito, timpinchile, amash, chile congo, mosquito chili, kipin, and chilpaya (13–16). This wild resource contains nutraceutical compounds integrated with phenolics, alkaloids, carotenoids, and volatile compounds. Piquin chili fruits are regionally consumed mainly through direct extraction from the ecosystem (17, 18).

The piquin chili represents a food resource that provides nutraceutical compounds to the diet of the rural population since consumption is associated with customs from the chili distribution area, thereby highlighting the interaction of identity and culture (17, 19). As a wild plant, it shows wide genetic variability and plasticity resulting from local adaptations (20). Hence, its nutritional and nutraceutical composition results from genetic influence, the environment in which it is grown, ecological interactions, harvesting practices, harvesting season, and post-harvest storage and processing (21). The research on the phytochemicals and nutraceutical potential of the wild forms of *Capsicum* is limited, compared with improved and commercial varieties of *C. annuum*, *C. frutescens*, and *C. chinense* that have been extensively studied (22–25). Although there are studies about the phytochemistry of piquin chili, management has yet to be archived to ensure the content of bioactive compounds with potential effects. The nutraceutical potential of piquin chili leads is influenced by nature and management conditions, supporting that holistic knowledge can reinforce future initiatives for establishing quality criteria for this spice focused on consumer benefits by means of chemical diversity, variation in contents, and evidence about accumulation patterns in wild and cultivated natural populations.

According to official statistics in Mexico^1^, the production of piquin chili is not as high as other varieties; however, the commercial value of a ton of cultivated green piquin chili is close to US$ 23,800. In comparison, a ton of jalapeño chili is approximately US$ 500, and a ton of serrano chili is approximately US$ 640. Regarding its role in the rural economy, it was recently highlighted that the price per kilogram of wild piquin chili is strongly associated with the number of fruits collected per day, which depends on multiple environmental factors and the season during which the plants are produced. For example, in April, in the northeastern region of Mexico, a kilogram of green wild piquin costs up to US$ 85. The wild piquin chili is considered a social identity resource where the collection is performed by the male gender given the remoteness and isolation of the wild piquin populations; however, the cleaning and selection of the fruits are carried out by women, which shows the relevance of the piquin chili in the territory (26).

## 2 The piquin is a wild source of nutraceuticals

**Figure 1:**
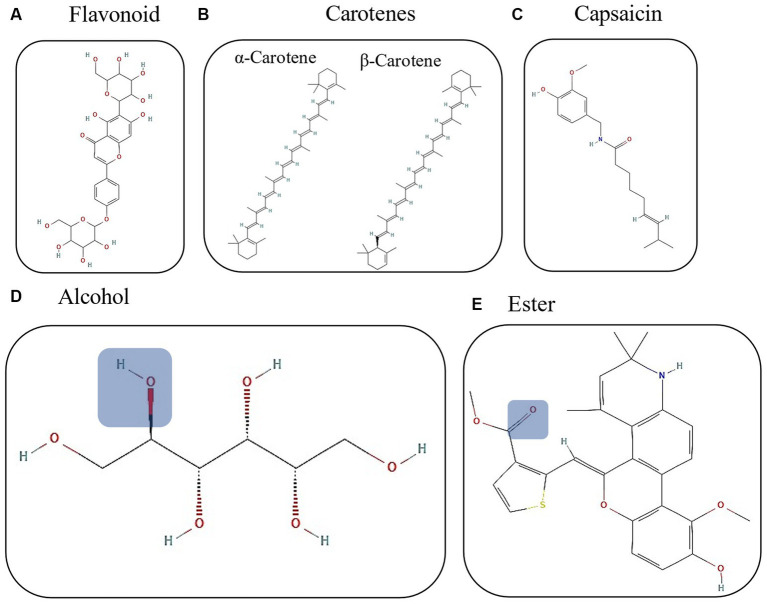
Chemical structures of the principal chili compounds. **(A)** Capsaicin (PubChem CID: 1548943); **(B)** α and β-carotenes (PubChem CID: 6419725 and 5280489); **(C)** flavonoid structure (PubChem CID: 73981632); blue squares represent the functional group for **(D)** alcohol (PubChem CID: 5780) and **(E)** ester (PubChem CID: 17758570). All chemical structures were reproduced from PubChem.

The dependence on plant resources to obtain functional benefits lies in the knowledge acquired through generations, traditional knowledge, and seasonal use related to the productive and phenological cycle of plants in native, indigenous, and rural communities (27, 28). The consumption of wild plants, such as piquin chili, has a close relationship between humans and the ecosystem; for example, this natural resource activates the local economy due to the interest of people to acquire piquin plants, fruits, and derivatives such as spicy oils, sauces, salts, and dry milled chili. All these products originate in rural areas and are sold in rural and cities, where consumers recognize them by their flavors and pungency (29, 30). As a wild species, its organoleptic and phytochemical characteristics vary (Figure 1), causing an impact on the consumer. Although agroforestry or greenhouse production programs exist, few species have achieved the phytochemical contents associated with the characteristics required by the consumer. It is essential to understand the impact of environmental conditions on the growth, organoleptic, and functional characteristics of the piquin chili due to its various compounds with nutraceutical potential that are highly influenced by the environment (Table 1) (20).

**Table 1:** The main phytochemicals identified in piquin chili.

**Phytochemical**	**Line, ecotype, and genotype**	**Content range**	**Variation source**	**Reference**
Flavonoids	Chiltepín Northwest Mexico	4.24 ± 0.10 mg/g DW	Nr	Hayano-Kanashiro et al. (14)
	Amashito Garbanzo	13.93–20.56 mg/g DW 6.15–10.32 mg/g DW	Phe	De la Cruz-Ricardez et al. (31)
	Cumpas Sahuaripa	2.34 ± 0.14–4.14 ± 1.0 mg CE/g DW 1.32 ± 0.17–3.53 ± 1.3 mg CE/g DW	Gr, Phe	Vázquez-Flores et al. (32)
	Amashito Garbanzo	9.01–11.56 mg QE/g DW 5.06–9.62 mg QE/g DW	Gn, Na	De la Cruz-Ricardez et al. (33)
	1S 15 Ct, 14 Ct	0.24, 0.22 0.36, 0.70	Gr	Moreno-Ramírez et al. (20)
Carotenoids	Cumpas Sahuaripa	1.60 ± 0.02–6.03 ± 0.08 mgβCE/g DW 0.66 ± 0.002–5.70 ± 0.8 mgβCE/g DW	Gr, Phe	Vazquez-Flores et al. (34)
	Chiltepín Northwest Mexico	12.81 ± 0.7–33.23 ± 0.3	Phe	Hayano-Kanashiro et al. (14)
	Amashito Garbanzo	≈5.0–≈10.0 mg/g DW ≈2.0–≈15.0 mg/g DW	Phe	De la Cruz-Ricardez et al. (35)
	Amashito Garbanzo	5.41–23.71 mg/g DW 3.71–28.8 mg/g DW	Phe, Ag	De la Cruz-Ricardez et al. (33)
Capsaicinoids	III-1, I-3	13.7 ± 0.1, 27.2 ± 0.3 mg/g DW	Na	Moreno-Ramírez et al. (18)
	Pue 01, Nay 02	135 ± 19 μg/ml, 1,379 ± 95 μg/ml	Na	Díaz-Sánchez et al. (36)
	Oax10, Ags01	301 ± 34 μg/ml, 3,719 ± 101 μg/ml	Gn	Díaz-Sánchez et al. (36)
	San Fernando	0.67 ± 0.37–3.13 ± 0.33 mg/g DW	Ag	Valiente-Banuet and Gutiérrez-Ochoa (37)
	Cumpas Sahuaripa	1.68 ± 01–8.73 ± 0.06 mg/g DW 1.41 ± 0.01–8.59 ± 0.04 mg/g DW	Gr, Phe	Vazquez-Flores et al. (34)
	G8	0.56–1.06 mg/g FW	Phe	Morales-Fernández et al. (38)
	Chiltepín (Querétaro, México)	216.22 ± 0.68–1249.28 ± 12.54 mg/g DW	Phe	Fayos et al. (39)

Phe, phenological state variation; Na, natural variation; Ag, variation due to agronomic management; Gn, genotype variation; Gr, variation for geographic origin; Nr, not reported; DW, dry weight; FW, fresh weight; CE, catechin equivalent; QE, quercetin equivalent; mgβCE, β-carotene equivalent.

## 3 Flavonoids

Flavonoids are specialized metabolites with a 15-carbon structural core called the flavone skeleton, where different chemical substitutions (Figure 1A) give them biofunctional and antioxidant properties. They are essential in the food, cosmetics, and pharmaceutical industries. Flavonoids can accumulate in specific organs or tissues in significant concentrations, which lead to identifying rich natural sources of these compounds (40). The presence of flavonoids in piquin chili has been repeatedly reported, highlighting its phytochemical potential as a natural source (18, 20).

The piquin chili flavonoid concentrations vary between the evaluated accessions. Samples collected in the areas of Valles Centrales, Sierra Sur, and Oaxaca Isthmus (Southern Mexico) had flavonoid concentration differences according to the immature and mature state, with 0.5 and 0.95 mg per gram of tissue, respectively, showing a difference close to 50% of flavonoid concentration. However, it does not consider the chemical diversity of total flavonoids (41). The mature chili peppers obtained in Sonora (Northwest of Mexico), analyzed through HPLC-DAD, reported piquin chili with 0.065 ± 0.006 mg per gram in dry weight (DW) (42). Mature chili peppers collected in the central, coastal, plain, and southern regions of Tamaulipas (Northeast of Mexico) had contents between 0.24 and 0.36 mg of quercetin equivalents per gram of tissue (20), lower than those reported in the Oaxaca samples (41). Among populations within a region, mature piquin chili fruits have flavonoid contents with variations that reach up to 30%. Even the content of flavonoids is higher than in some chili-cultivated varieties (20, 43, 44). This highlights the precedence of the germplasm and its ripening process to maximize flavonoid contents and unleash the full range of potential benefits associated with consuming these phytochemicals (45). In this regard, the variation in flavonoid contents in several chili species and genotypes has shown dynamic patterns, where the environmental conditions have been remarked as more important in the final contents; however, the genetic interaction, agronomical practices, and the direction of particular flavor by artificial selection according to preferences and uses should not be discarded to reach high yields when the purpose is harvesting chili fruits rich in flavonoids (22, 46).

## 4 Carotenoids

Carotenoids are lipophilic compounds produced by plants that protect photosynthetic systems from light excess and are precursors of phytohormones. The carotenoids have a 40-carbon skeleton derived from repetitive condensation of isoprene units (Figure 1B) and have been considered of nutraceutical interest against complications associated with oxidative stress (47). For the chili peppers, carotenoids contribute to the coloration of the mature states, which is evident as the chlorophyll degrades (48, 49). In this regard, it is worth highlighting the marked influence of environmental conditions and the genetics of chili peppers regarding the accumulation of carotenoids, which have been regularly identified in various chili genotypes, where specific accumulation patterns have been observed (50–52). As an example, in the piquin chili, the carotenoid content has been reported as contrasting between the immature (green fruit) and mature states (red fruit), in addition to being undetectable in the first state and reaching considerable levels when ripe (33).

However, its accumulation and degradation patterns are highly dynamic at a molecular level, which results in different amounts and a wide chemical diversity of carotenoids and related compounds during the ripening process (51). While it is suggested that the maximum accumulation of some carotenoids is reached when the chilies mature, this process is responsible for the bright and attractive tones of the chilis (53). Mature chili is recommended for use due to its accumulation of some carotenoids and its increased antioxidant property, which is associated with its industrial uses and consumption to obtain the greatest nutraceutical effects (32). The Cumpas and Sahuaripa chilies, ecotypes of piquin chili, showed levels with significant differences of β-carotene on mature chilies, with 6.03 and 5.70 mg per gram for DW, respectively. Both chilies were from Sonora State (northwest of Mexico) and were grown under greenhouse conditions (34). Two ecotypes of piquin chili from Tabasco State (southeastern Mexico) showed significant differences between carotenoid contents in ripe and unripe fruits. Mature fruits show higher carotenoid content and are subjected to various levels of shading, reaching up to 28.80 mg g^−1^ of DW in the dry season, in an open sky system for the Garbanzo genotype and 23.71 mg g^−1^ of DW in the rainy season, in an open sky system for the Amashito genotype (50). The same research shows a difference in carotenoid levels in mature chili peppers, and these differ according to the shading conditions, with higher levels when the plants are exposed to natural light in the open field system and lower levels when shade conditions are increased. The carotenoid levels were different between seasons and humidity. The amount of pigment, associated mainly with carotenoids, present shows a wide heterogeneity between ecotypes of piquin chili from a wide area sampled in Tamaulipas (northeast of Mexico) (20). Thus, the levels of carotenoids present in piquin chili are highly influenced by phenological, genotypic, and environmental conditions, which increase the complexity of the consensus under which this wild plant can have a specific content and stability according to its growth conditions. Although this could be considered a disadvantage for intensive or industrial use, it represents an opportunity to strengthen local uses, which take advantage of this heterogeneity to increase added value for local consumers.

## 5 Capsaicinoids

Capsaicinoids (CAPs) are compounds synthesized mainly in the pericarp and placental tissue of chili fruits (*Capsicum*) and are considered within the group of alkaloids. This biosynthesis is highly controlled at a molecular level (54, 55). *Capsicum* plants synthesize them as a defensive mechanism against herbivores and phytopathogens. The CAPs show low polarity and are structurally based on a vanilloid group (an aromatic ring with a hydroxyl and a methyl group), together with a long hydrocarbon chain and the amide group (Figure 1C) (56, 57). The study of CAPs aims to optimize the content of chili fruits, especially the capsaicin responsible for chili’s heat and pungency. Effects such as antitumor, antiangiogenic, antineoplastic, thermogenic, and antimicrobial have been related to CAPs (57, 58). It also relates to industrial applications such as functional coatings, clinical applications, food uses, and biotechnological developments (59, 60). Therefore, several investigations address ideal conditions, biosynthesis, and extraction methods with optimal yields, low prices, high purity, and concentration to meet market demands. In this regard, the accumulation of capsaicinoids in chili fruits depends on the growing conditions, climate, genotype, and agronomic management (61, 62). The biosynthesis of capsaicinoids presents a highly dynamic genetic activity during fruit development, and there is evidence that supports changes in pungency driven during the domestication process in the case of *C. annuum*, being different between wild types and cultivated relatives (63). The accumulation of capsaicinoids in *C. annum* var. *annum* has been characterized, indicating that approximately 90 days after anthesis, the fruits reached the highest content. This information highlights the appropriate time for harvesting chili fruits when capsaicinoids are the compounds of interest (31).

For piquin chili, the CAP content evaluations present a wide variation. The content of capsaicin and dihydrocapsaicin under wild conditions is not affected once the fruit has reached its size of commercial interest, being statistically similar between mature and immature fruits (35); however, there is evidence that levels of capsaicinoids could change, the highest level being discovered in mature fruits and when they are cultivated under greenhouse conditions (36). The proportion of capsaicin and dihydrocapsaicin in 16 wild native populations presented similar levels, but there was observed variation in capsaicinoids per population sample (20). Díaz-Sánchez et al. (36) examined 31 accessions of piquin chili that are grown under greenhouse conditions with similar capsaicin and dihydrocapsaicin ratios. Variations in the total content of capsaicinoids were shown, with the lowest value being 301 ± 34 in the Oax10 accession and the maximum value being 3,719 ± 101 μg/ml in Ags01 (36). During the process of fruit formation and ripening, a high classification of both capsaicinoids has been established, which indicates that these proportions are maintained even when the ripening time varies (39). The number of capsaicinoids could be maintained without variation when the growth conditions are not so drastic or long-lasting as to impact the accumulation of these metabolites in the fruit (64). However, the content of total capsaicinoids has been reported with differences according to the degree of ripening under controlled conditions, with 1.41 and 1.68 mg g^−1^ DW in the immature state and 8.59–8.73 mg g^−1^ DW in mature fruits of the Sahuaripa and Cumpas lines, respectively (34). The analysis of ripe fruits of wild piquin chilis from Tamaulipas showed that the contents of total capsaicinoids were statistically different and showed differences among ecotypes in the same municipality (18, 65). The above result suggests that diverse environmental factors may influence the final accumulation of total capsaicinoids more than other factors. Due to this, it is suggested that knowledge of ideal environmental conditions could support the selection of sites with the best characteristics for collecting fruits with the highest CAP contents (65) for diverse purposes, including their biosynthesis, their ecophysiological role, and the promotion of regional or international marketing. Recently, it was demonstrated that the accumulation of CAPs in piquin chili also follows a pattern similar to other chili varieties, which could help in breeding programs to obtain more pungent chilis and to establish times for collecting fruits with optimal contents (66).

All previous studies support the relevance of understanding the impact of growing conditions and genetic components, which influence piquin fruits with specific phytochemical contents. Although some initiatives have been made to stimulate agroforestry and greenhouse production, the yields and qualities are hard to emulate, mainly due to the impact of genetic diversity and multifactorial environmental influences; hence, the more we learn, the closer we get close to improving the management of this species.

## 6 Volatile compounds

In chili, this category includes a heterogeneous group of chemical compounds (Figures 1D,E) responsible for aroma and partial flavor, which influences the organoleptic perceptions of consumers and criteria for specific uses (67, 68). They are organic, low molecular weight compounds, and volatile at environmental temperatures (69). In *Capsicum* species, several chemical groups have been reported, including aldehydes, organic acids, esters, lipoxygenase cleavage products, nitrogen-containing compounds, hydrocarbons, alcohols, ketones, furans, terpenoids, phenolics, and miscellaneous compounds, highlighting the complexity of volatile compounds in these fruits. However, not all compounds contribute to the aroma, and chemical diversity fluctuates between species, varieties, and phenology (68, 70). Piquin chili is also preferred by consumers due to its aromatic traits, especially in the green stage, with fresh, fruity, and herbal notes, which are associated with its complex chemical array. Piquin chili includes up to 140 compounds, among which esters, alcohols, aldehydes, ketones, terpenes, organic acids, and hydrocarbons were detected (71). This wild chili shares some volatile compounds responsible for fruity notes with *C. chinense* Jacq. cv. Habanero and *C. frutescens* L. (72, 73). The volatile compounds in piquin chili change during the ripening process, with the high diversity and contents in the green stage decreasing as it matures in the case of esters; however, mature fruit shows slightly higher levels of other compounds. This suggests that flavor and aroma are different, influencing consumer preferences for uses depending on their ripening stages (71).

All this evidence supports the potential of the piquin chili to provide phytochemicals associated with benefits for human health; however, its consumption is driven by cultural issues linked to traditional uses and forms of preparation that influence the intake of these compounds. In this regard, the purpose of the consumption and the traditional use must be considered and linked with all information about the chemical composition in different stages. As mentioned above, the phytochemical groups explored in this study showed great variation as a result of multifactorial influences (genetic background, highly dynamic regulation of biosynthesis, environmental effects, and agronomic issues), which makes it challenging to designate ideal conditions for collecting chili fruits from the wild. Nevertheless, cultivated piquin chili was shown as a better strategy to produce fruits with more stable phytochemical contents to benefit consumers and also with the potential to reduce the extraction pressure in natural populations. Therefore, developing strategies to improve agronomic management to increase the availability of chili with better phytochemical contents derived from optimal growing conditions and versatile genotypes is needed.

## 7 Conclusion

Since piquin chili is a wild spice obtained from natural populations, and in some cases with incipient agronomic management, holistic studies of the piquin chili should be considered to understand the factors that affect the biosynthesis of the compounds responsible for the organoleptic properties and bioactive components to guarantee the ideal contents for the benefit of the consumer, which will strengthen its trade at a local and international level, in addition to promoting the conservation of this resource. This requires genetic knowledge, agronomic knowledge, and technological improvements focused on the integrative management of piquin. Piquin chili is not only considered for its commercial or nutraceutical value but also for its holistic knowledge relevant to being the ancestor of many cultivated varieties of commercial chili peppers, making it a basis for understanding and improving the production of such varieties. On the other hand, piquin domestication focuses on obtaining lines that satisfy consumers or market requirements in specific ways to reduce extraction pressure on natural populations. However, what highlights its significance is the relationship since its recollection and exploitation as a wild resource, linked to customs, regional uses, and biocultural factors.

## Author contributions

RP-R: Conceptualization, Validation, Writing – original draft, Writing – review & editing. YM-R: Validation, Writing – original draft, Writing – review & editing. GR-D-L-C: Writing – original draft, Writing – review & editing. MJ-A: Investigation, Writing – original draft, Writing – review & editing. CA-M: Investigation, Writing – original draft, Writing – review & editing. NN-G: Writing – original draft, Writing – review & editing. JT-C: Conceptualization, Investigation, Supervision, Writing – original draft, Writing – review & editing.

## Footnotes

^1^https://nube.siap.gob.mx/cierreagricola/ (Accessed March 22, 2024).
